# Tobacco Smoke in Strangulated Hernia

**Published:** 1852-06

**Authors:** 


*Tobacco Smoke in Strangulated Hernia.*

Dr. F. J. Stratton, of West Alexandria, Ohio, mentions a case in a communication in the *Western Lancet*, in which tobacco-smoke was employed by him in strangulated hernia. The subject was a young man of robust constitution and athletic frame, about twenty-three years of age. The protruded intestine resisted prolonged efforts at reduction by the taxis, the use of opiates and free venesection. Chloroform could not be employed on account of its unpleasant effect on the patient.

Dr. S. then prepared to operate, but, as a last resort, concluded to try the effect of throwing tobacco-smoke up the rectum. This was done by making a connection between the stem of a pipe, and a large-sized gum-elastic catheter, the latter being passed up the rectum. The bowl of the pipe being filled with strong tobacco, and ignited, an individual applied his mouth over it, and blew a large volume of smoke in. "In three minutes the patient complained of nausea and a desire to evacuate his bowels," after which the reduction was easily effected, and was followed by no unpleasant consequences.

